# A reconstruction theory of relational schema induction

**DOI:** 10.1371/journal.pcbi.1008641

**Published:** 2021-01-26

**Authors:** Steven Phillips

**Affiliations:** Human Informatics and Interaction Research Institute, National Institute of Advanced Industrial Science and Technology, Tsukuba, Ibaraki, Japan; Durham University, UNITED KINGDOM

## Abstract

Learning transfer (i.e. accelerated learning over a series of structurally related learning tasks) differentiates species and age-groups, but the evolutionary and developmental implications of such differences are unclear. To this end, the *relational schema induction* paradigm employing tasks that share algebraic (group-like) structures was introduced to contrast stimulus-independent (relational) versus stimulus-dependent (associative) learning processes. However, a theory explaining this kind of relational learning transfer has not been forthcoming beyond a general appeal to some form of structure-mapping, as typically assumed in models of analogy. In this paper, we provide a theory of relational schema induction as a “reconstruction” process: the algebraic structure underlying transfer is reconstructed by comparing stimulus relations, learned within each task, for structural consistency across tasks—formally, the theory derives from a category theory version of *Tannakian reconstruction*. The theory also applies to non-human studies of relational concepts, thereby placing human and non-human transfer on common ground for sharper comparison and contrast. As the theory and paradigm do not depend on linguistic ability, we also have a way for pinpointing where aspects of human learning diverge from other species without begging the question of language.

## Author summary

Learning transfer—an improvement in the rate of learning over a series of learning tasks—differs between species and age-groups, but the implications of such differences are unclear. Relational schema induction is a form of learning transfer that highlights relational aspects of learning. However, a theory explaining this kind of induction and transfer has not been forthcoming beyond a general appeal to some form of analogical mapping, as typically assumed in models of analogy. I present a theory of relational schema induction as a “reconstruction” process: the common structure affording transfer is reconstructed by comparing stimulus relations learned within each task for structural consistency across tasks. The theory also applies to non-human studies of relational concepts, thereby placing human and non-human studies on common ground for a clearer comparison and contrast.

## Introduction

Learning transfer refers to an improvement in the rate of learning across a series of structurally related tasks [1]. For example, suppose each task is to learn a configural association, where the target responses depend on interacting cues (colour and shape): in one task instance, subjects are trained to select square (triangle) when presented with a square (triangle) on a blue coloured background, and triangle (square) when presented with a square (triangle) on a green background; in another instance, subjects are trained to select circle (cross) when presented with a circle (cross) on a red background, and cross (circle) when the background for the presented circle (cross) is yellow. Evidence of learning transfer is observed when the number of training trials needed to reach criterion decreases on subsequent task instances.

Many species have a capacity to learn, yet its unclear whether differences in learning transfer are quantitative, or qualitative [2–4]. For instance, some authors have argued for an association-based (stimulus-dependent) account of learning that is extendable to propositions, so providing a basis for higher cognition [5]. An associative model could assume that learning rate changes with prior experience [6], thereby providing a quantitative explanation for differences in learning transfer: greater transfer is linked to greater change in learning rate. Yet, other authors have argued that the propositional (relational) aspects of cognition—inferring targets from stimulus-independent relations—are qualitatively unique to humans [7], and most developed in adults [8]. Resolving such disputes over accounts of learning transfer should inform the nature, evolution and development of cognition.

To this end, the *relational schema induction* paradigm was developed to distinguish associative versus relational models of learning transfer, in human studies [9, 10]. These models make contrasting predictions following feedback on *information trials* that can be used to determine the responses to the other stimuli. For example (above), having seen that circle is preferred over cross when a circle is presented on a red background (information trial), a relational model predicts that cross is preferred over circle when the background is yellow, because the second task involves the same relation(al schema). By contrast, the associative model makes no prediction for a novel stimulus, having not been paired with a target before. The data for various instances of this paradigm support a relational model for learning transfer [9, 10].

Relational schema induction is suitable for comparing/contrasting species and age groups, because the paradigm does not require language to administer. However, a theory explaining this kind of learning transfer has not been forthcoming beyond a general appeal to some form of structure-mapping [9, 10], as typically assumed in models of analogy [11, 12]. Such theory would help clarify the extent to which evidence of relation-based transfer in other species is comparable to that observed in humans.

We develop a theory of relational schema induction that is applicable to non-human studies to help redress this situation. The motivation for our approach is two-fold: (1) the observation that relational schema induction is a form of *Tannakian reconstruction* [13], and (2) the fact that Tannakian reconstruction is a *universal construction* in the category theory sense [14]. In this way, our theory derives from a category theory version of Tannakian reconstruction. Briefly, relational schema induction involves a sequence of cue-target learning tasks where the cues can be reinterpreted as (permutation) actions on a set of stimuli: e.g., shapes sending trigrams to trigrams, which constitute a permutation representation of a task. The actions constitute a group-like (algebraic) structure that is reconstructed from the within-task and between-task trigram relations. Reconstruction obtains by computing a universal construction, called the *end of a bifunctor* [14].

Our category theory approach is also motivated by way of explaining a *systematicity* property [15] with regard to relational schema induction, i.e. why a capacity for relational schema induction in one situation implies a capacity for relational schema induction in another situation. Universal constructions explain systematicity properties [16]: certain equivalences between cognitive capacities—formally, capacity *A* if only if capacity *B* [17]. The end of a functor is a universal construction. Hence, our category theory approach also accounts for the systematicity of relational schema induction.

We proceed by presenting an example of the relational schema induction paradigm and background category theory in the rest of the Introduction section. The reconstruction theory for relational schema induction is developed and applied in the Results section, where it is also shown to account for other examples of relational schema induction from the literature [9, 18]. Implications of the theory are explored in Discussion and theoretical details, including definitions, examples and theorems, are provided in Methods. The main theorem is a known result; the application of this theorem to cognition appears to be new.

### Relational schema induction paradigm

**Fig 1:**
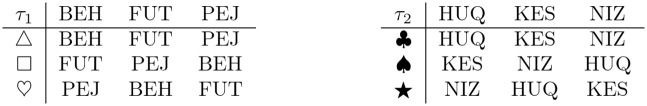
Two task instances, *τ*_1_ (left) and *τ*_2_ (right), for the relational schema induction paradigm, specified as “multiplication” tables.

In a relational schema induction experiment [9] (experiment 2), participants were administered a series of cue-target learning tasks. Each task required participants to learn a map from a set of (shape, trigram) pairs to a set of trigrams. For instance, suppose stimuli were drawn from the set of shapes *Sh* = {△, ☐, ♡} and the set of trigrams *Tri* = {BEH, FUT, PEJ}, where the map from cues to targets, *τ*_1_: *Sh* × *Tri* → *Tri*, is given by a “multiplication” table (Fig 1, left): e.g., the pair (△, BEH) maps to target BEH, the pair (☐, BEH) maps to FUT, and so on. Hence, participants were required to learn nine cue-target mappings. Learning was afforded by feedback, indicating the target, following their response to each cue. After learning the task, a new learning task was administered, *τ*_2_, where stimuli were drawn from another set of unique shapes and trigrams (Fig 1, right). Each task instance involves the same group-like structure, where the shapes constitute the actions on the set of trigrams. Four learning tasks were administered and performance in terms of correct responses was recorded.

**Fig 2:**
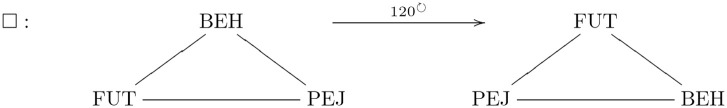
A cyclic-3 group action on a set of trigrams as a rotation.

The actions have a geometric interpretation by assigning trigrams to the vertices of a triangle: 0°, 120° and °240° (equivalently, -120°) rotation. For example, ☐ (first task) corresponds to a clockwise rotation that sends BEH to FUT (Fig 2), i.e. the left triangle rotated 120° to obtain the right triangle. Formally, rotation is the (cyclic-3 group) action ↻: ℤ/3ℤ×Tri→Tri, and a 120° rotation is the map ↻: (1, BEH) ↦ FUT, (1, FUT) ↦ PEJ, (1, PEJ) ↦ BEH.

Note that the particular ordering of trigrams and shapes given in this example is for expository purposes. Swapping the columns (or, rows) of the multiplication table does not change the effect of the action: e.g., ☐ : FUT → PEJ could be interpreted as saying that PEJ “follows” FUT, or that PEJ “precedes” FUT. The way that shapes are interpreted as acting on trigrams, however, must be consistent throughout the task and with the given cue-target mappings to afford induction and learning transfer. The paradigm and our ensuing theory do not depend on assuming a particular ordering for trigrams, or shapes.

The empirical results of interest here are the number of first-trial response errors for each task instance in the *consistent* condition, i.e. where all task instances conform to the same structure (see [9], p. 224), as an indicator of induction and learning transfer. Participants were not informed of the group-like nature of the tasks, so first-trial responses for the first task were expected to be at chance level. However, induction affords learning transfer given feedback on information trials that allows participants to correctly predict targets for the other seven cues, assuming the trials involved two different rotations. For example, suppose feedback from two information trials that identify the mappings (♠, KES) ↦ NIZ and (★, HUQ) ↦ NIZ, which implies that ♠ and ★ correspond to the two (non-zero) rotations. The remaining cues are inferred by completing the rest of the multiplication table. Note that if the two information trials involve the same rotation action, then another information trial involving a new shape is needed to determine the actions of the other shapes. If the cues are selected without replacement, the number of information trials needed is between two and four, depending on the distribution of cues. The observed average number of first-trial errors was six for the first task, which corresponds to chance level (i.e. two-thirds of nine trials), and less than three for the third and fourth tasks [9], which is consistent with the two information trials (assuming distinct shapes) needed for transfer.

### Basic category theory

Category theory constructions reside in a *category* (definition 1) of some kind, so the first step towards a categorical theory of relational schema induction is to treat the induction paradigm as a category, which means specifying the constituent *objects*, *morphisms* and *composition operation*. Morphisms can be regarded as (directed) relations between objects, and the collection of such relations from an object to an object is called a *hom-set* (remark 2). The collections of sets and functions between sets form a category, denoted **Set** (example 3). Relational schema induction involves a series of related (learning) tasks, so we regard the induction paradigm as a category of tasks (objects) and task relations (morphisms) to be specified next.

The objects are specified as follows. Each learning task, *τ*_*i*_: *Sh* × *Tri* → *Tri*, is equivalently constituted of actions (shapes) on a set (of trigrams). The set of actions has the algebraic structure of a *monoid* (definition 5), i.e. a set together with a *binary operation*, called “multiplication,” and a special element, called the *unit*. Monoids abstract a familiar situation in elementary arithmetic (example 6): e.g., the integers together with (elementary) multiplication and one, as the unit, form a monoid. Recall that the elements of the set of shapes *Sh* = {△, ☐, ♡}, for the task instance *τ*_1_, correspond to rotations of a triangle. These actions constitute the monoid *M* = (*Sh*, ⋅, △), where the binary operation is multiplication of actions (e.g., ☐ ⋅ ☐ = ♡) and △ is the unit (e.g., △ ⋅ ☐ = ☐ = ☐ ⋅ △). As monoid actions on a set, the learning task *τ*_1_ is formally a *(left) monoid action* (definition 10) on the set of trigrams *Tri* = {BEH, FUT, PEJ}. The monoid action, *τ*_1_, is also called an *M-act*, and the set acted on, *Tri*, is called an *M-set*—together, the pair (*Tri*, *τ*_1_), denoting a particular task, is called an *M-set representation* (definition 10). Each task has the same monoid structure, hence the collection of (possible) task instances constitutes the objects (M-set representations) for our category of (learned) tasks. The monoid in this example is equivalent to the integers with addition modulo-3, which is also a group (remark 7), i.e. a monoid where every element has an inverse.

The morphisms in our category of tasks specify the “action-compatible” relations between tasks, called *equivariant maps* (definition 11). The composition operation is composition of equivariant maps. In the current example, an equivariant map is an assignment of the trigrams in one task to trigrams in another task that is compatible with the associated actions for *τ*_1_ and *τ*_2_. Compatibility essentially means that applying the map to an action is the same as applying the action to the map. The map *f*: BEH ↦ HUQ, FUT ↦ KES, PEJ ↦ NIZ is an equivariant map: e.g., *f*(*τ*_1_(☐, FUT)) = NIZ = *τ*_2_(♠, *f*(FUT)). One can think of an equivariant map as an analogy between tasks. For a monoid *M*, the collection of (possible) tasks and equivariant maps between tasks forms a category, denoted **MSet** (remark 12). A map that has an *inverse* is called an *isomorphism* (definition 13), which plays an important role in our category theory approach to relational schema induction.

Most models of cognition assume some kind of function modeling information about the world that gets presented as input to the cognitive system. A category theory version of this situation is a *functor* (definition 18) from a category modeling the world (e.g., the experimental setting) and a category modeling what the participant sees. Cognitive models typically regard cognitive processes in terms of functions between sets of cognitive states. Sets and functions constitute objects and morphisms in the category **Set**. Accordingly, the relationship between experiment and participant is a functor from the category **MSet**, modeling the experimental paradigm, to the category **Set**, modeling cognitive states of the participant. An important aspect of the induction paradigm is that the monoid structure of the tasks is not (explicitly) communicated by the experimenter to the participant. Rather, this structure is induced from the stimulus relations defining the learning tasks. This situation is given by the *underlying/forgetful functor* (example 22), *U*: **MSet** → **Set**, which sends each task (M-set representation) to its underlying set of trigrams, forgetting the actions, i.e. *U*: (*S*, *σ*) ↦ *S*, where *σ* is the monoid action on the set *S*. For example, *U* applied to the M-set representation of task *τ*_1_ yields the set of trigrams {BEH, FUT, PEJ}. The equivariant maps are sent to the functions between corresponding sets. In other words, the participants only “see” the *image* (definition 26) of the forgetful functor, not the monoid structure from which the tasks were constructed, and they “remember” (learn) the actions as (invertible) maps on sets of trigrams.

**Fig 3:**
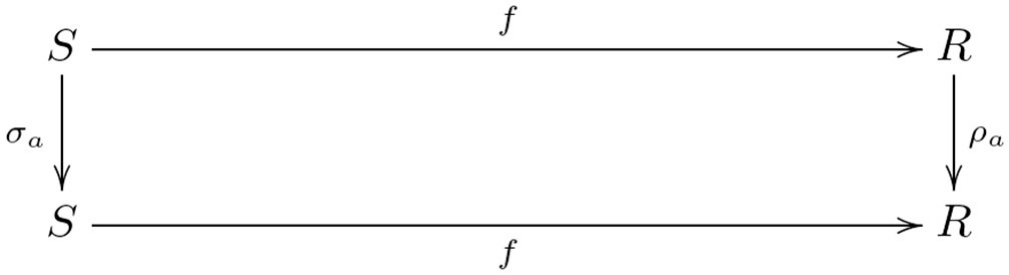
The image of the forgetful functor includes within-task trigram relations (vertical arrows), i.e. the action *a* on the set of trigrams *S* (*R*) is the function *σ*_*a*_ (*ρ*_*a*_), and between-task trigram relations (horizontal arrows), i.e. equivariant map *f* from *S* to *R*.

The image of the forgetful functor includes both within-task and between-task trigram relations (Fig 3). The within-task relations arise directly from learning the mappings from trigrams to trigrams for each shape and task. The between-task relations arise indirectly, as the maps satisfying a *commutativity* condition (remark 27), since each task instance is administered separately: by recognizing that a map *f* of a trigram *t* in a set of trigrams *S* (constituting one task) to the trigram *f*(*t*) in the set of trigrams *R* (constituting another task) followed by an action *ρ*_*a*_, i.e. *ρ*_*a*_(*f*(*t*)), is the same trigram as the action *σ*_*a*_ on *t* followed by the map *f*, i.e. *f*(*σ*_*a*_(*t*)) = *ρ*_*a*_(*f*(*t*)). Recognizing these commutative relations between the learned tasks is crucial to inducing the monoid. Formally, a comparison of images (functors) is a *natural transformation*, i.e. a map between the objects and morphisms of the images that satisfies a commutativity (naturality) condition (definition 28). In certain situations of interest here the comparison is bidirectional, i.e. a *natural isomorphism* (definition 30).

## Results

With the background category theory in place, we now develop our reconstruction theory of relational schema induction and apply the theory to account for the data given in the previous section. The theory does not depend on the specific details of this example, which is shown by application to other examples of relational schema induction in the second part of this section.

### Reconstruction theory

Our reconstruction theory of relational schema induction derives from a category theory version of Tannakian reconstruction [13], whereby the monoid, *M*, is reconstructed from the category of learning tasks (M-set representations), **MSet**, via the forgetful functor, *U*. The theory says that *M* is recovered from a particular kind of category theory construction involving *U* (theorem 65). Monoid *M* obtains from an “optimal” comparison of trigram relations within and between tasks, i.e. the actions and equivariant maps, respectively. A description of the theory is given in two stages: the first stage concerns the induction of the monoid (Induction as reconstruction section), and the second stage concerns the application of the induced monoid for learning transfer on new tasks (Transfer as completion section).

#### Induction as reconstruction

Induction involves an optimal comparison of within-task and between-task trigram relations. A description is given in three parts to facilitate an understanding of this construction and its correspondence to putative psychological processes. The first part (Relational comparison) and the second part (Optimal comparison) pertain to the theory, i.e. the comparisons of trigram relations and the sense in which the comparisons are optimal, respectively. The third part pertains to a computational process for determining the optimal comparison, which corresponds to the relational schema (Schema computation).

#### Relational comparison

Comparison of trigram relations involves a *hom-functor* (definition 35) to determine within-task and between-task trigram relations. A hom-functor, Hom_**C**_(*A*, −): **C** → **Set**, determines the relationships of the objects and morphisms in a category **C** relative to (“landmark”) object *A*. In particular, for an object *X* in **C** the application Hom_**C**_(*A*, *X*) returns *efferent* relationships for *A* in the form of the hom-set of morphisms from *A* to *X*; for a morphism *f*: *X* → *Y* in **C** the application Hom_**C**_(*A*, *f*) returns the (*f*-)relations between the morphisms from *A* to *X* and the morphisms from *A* to *Y* in the form of an operation that sends each morphism *g*: *A* → *X* to the morphism *f* ∘ *g*: *A* → *Y*. Dually, ${\text{Hom}}_{{\text{C}}^{\text{op}}}(-,A)$ determines the *afferent* relations for *A*. By prefacing these hom-functors with the forgetful functor, *U*, we obtain relations between trigrams within and between tasks. Within-task relations are obtained by applying the same task: e.g., Hom(*U*(*S*, *σ*), −) applied to *U*(*S*, *σ*), which returns the hom-set of relations (including the actions) on trigrams in *S*, i.e. Hom(*S*, *S*). Between-task relations are obtained by applying a different task: e.g., Hom(*U*(*S*, *σ*), −) applied to *U*(*R*, *ρ*), which returns the hom-set of relations (including the equivariant maps) from the set of trigrams *S* to the set of trigrams *R*, i.e. Hom(*S*, *R*). For a psychological interpretation, the image of the hom-functor Hom(*S*, −) can be regarded as a cognitive map of trigrams relative to the focus of attention *S*.

We instantiate *S* and *R* to be the tasks specified by *τ*_1_ and *τ*_2_ (Introduction) to illustrate these comparisons. Suppose *S* (*τ*_1_) is our landmark task. The possible within-task trigram relations obtain from the application of Hom_**MSet**_(*U*(*Tri*, *τ*_1_), −) to (*Tri*, *τ*_1_), which is the hom-set, Hom(*Tri*, *Tri*), of possible functions on the set of trigrams {BEH, FUT, PEJ}. This set includes the actions of the shapes △, ☐ and ♡, which we label as the components *σ*_△_, *σ*_☐_ and *σ*_♡_, respectively. Likewise, the possible between-task trigram relations obtain from application of Hom_**MSet**_(*U*(*Tri*, *τ*_1_), −) to the second task yielding the set of possible functions from the trigrams in the first task to the trigrams in the second task. This set includes the equivariant map *f*: BEH ↦ HUQ, FUT ↦ KES, PEJ ↦ NIZ (see Introduction).

Relational comparison involves a comparison of trigram relations between tasks. So, we require a bivariate form of the forgetful function, i.e. a functor that takes two arguments that are the tasks whose trigram relations are being compared. A functor that takes two arguments is called a *bifunctor* (definition 40). Hence, relational comparison involves the functor Hom(*U*−, *U*−). Bifunctors are compared by a *dinatural transformation* (definition 44), which can be regarded as an analogous two-dimensional generalization of natural transformation between ordinary functors (remark 45). The dinatural transformation of interest here is a *wedge* (definition 46) to the bivariate form of the forgetful functor, written $\omega :D\ddot{\to }\text{Hom}(U-,U-)$. A wedge satisfies a commutativity (dinaturality) condition (diagram 7). For the current situation, this condition requires a set of elements *D* and a pair of functions (*ω*_*S*_, *ω*_*R*_), for each pair of learned tasks (*S*, *R*), that pick out the comparable within-task trigram relations. Within-task relations are comparable if they are equivariant, and the wedge identifies this condition as equality of the *di*agonal morphisms in the commutative diagram for the equivariance condition—there are two diagonal morphisms obtained by clockwise and anticlockwise traversal of the square of morphisms in Fig 3. In other words, each element in *D* picks out an action *σ*_*a*_ on the trigrams in *S* *and* an action *ρ*_*b*_ on the trigrams in *R* such that the equivariant map *f*: *S* → *R* composes with those actions to yield the same (“diagonal”) relation from *S* to *R*, which says that *ρ*_*b*_ ∘ *f* = *f* ∘ *σ*_*a*_. Psychologically, one can interpret this situation as finding an analogical mapping between tasks, though the analogy need not be a one-to-one correspondence, as typically assumed in analogy models [12], because an equivariant map is not required to be an isomorphism.

**Fig 4:**
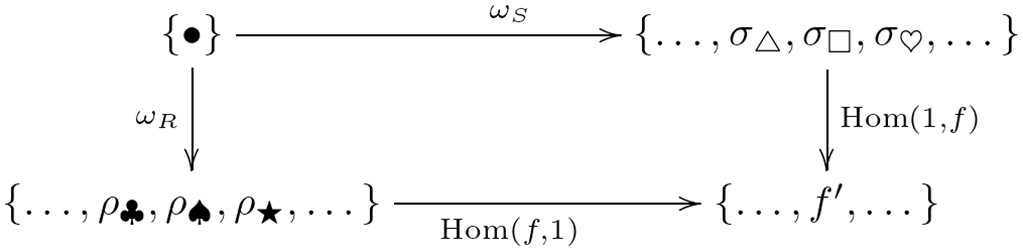
An example of relational comparison.

Continuing with the illustration, suppose *D* is the singleton set {•}, and *ω*_*S*_ and *ω*_*R*_ are the maps picking out the within-task trigram relations for each task. For the equivariant map *f*: BEH ↦ HUQ, FUT ↦ KES, PEJ ↦ NIZ, the (di)naturality condition requires a pair of action components such that composition with *f* yields the same map, *f*′, from the set of trigrams constituting the first task to the set of trigrams constituting the second task, as shown in Fig 4: e.g., *ρ*_♠_ ∘ *f* = *f*′ = *f* ∘ *σ*_☐_. In this instance, *ω*_*S*_ and *ω*_*R*_ pick out components *σ*_☐_ and *ω*_♠_, respectively.

#### Optimal comparison

Reconstruction involves determining which (sets of) comparisons are optimal, in a category theory sense to be explained shortly. Wedges determine comparable relations. A special wedge, called the *end* (definition 47), is optimal in the category theory sense of being *universal* (remark 48). In category theory, an optimal (universal) construction is formally defined as a *universal morphism* (definition 49). Thus, a universal wedge (end) is a particular kind of universal morphism. A universal morphism is optimal in being a common “point of reference” (in an abstract setting) for a family of related constructions. In the current concrete setting, the family of related constructions is the series of learning tasks and the common point of reference is the underlying monoid from which those tasks were constructed. An end is an optimal comparison of trigram relations, which consists of just those elements that are necessary and sufficient to pick out the actions of the monoid. These elements compose with the same monoid structure (theorem 65). Hence, computing the end of the bifunctor form of the forgetful functor reconstructs the underlying monoid, i.e. *∫*_**MSet**_ Hom_**Set**_(*U*−, *U*−) ≅ *M*. Ends are universal constructions, which are *unique up to unique isomorphism* [14], meaning that there is one and only one isomorphism satisfying commutativity (remark 52), so this monoid is essentially the same as the monoid formed by relabeling the two actions as particular shapes.

The sense in which an end is optimal is illustrated with the following two examples. The wedge (*D*, *ω*), where *D* = {•} is not an end, because it fails to satisfy the existence condition. The two-element set *D*′ = {•, *} also constitutes a wedge, (*D*′, *ω*′), that picks out two components in each task, i.e. $\omega_{S}^{\prime }:•\mapsto \sigma_{☐},*\mapsto \sigma_{♡}$ and $\omega_{R}^{\prime }:•\mapsto \rho_{♠},*\mapsto \rho_{★}$. However, this wedge does not *factor* through *D*, i.e. there does not exist a map *D*′ → *D* that composes with *ω* yielding *ω*′, as required by the univerality (unique-existence) condition for ends. In terms of the commutative diagram 8 for an end (definition 47)—our putative end *D* and wedge *D*′ correspond to objects *E* and *Z* (respectively)—there does not exist a morphism *u* such that *ω*′ = *ω* ∘ *u*. Conversely, the wedge (*D*″, *ω*″), where *D*″ = {⋄, •, *, ∘} and *ω*″ includes the mappings of *ω*′ and the mappings $\omega_{S}^{\prime \prime }:⋄\mapsto \sigma_{△},\circ \mapsto \sigma_{☐}$ and $\omega_{R}^{\prime \prime }:⋄\mapsto \rho_{♣},\circ \mapsto \rho_{♠}$, is also not an end. In this case, the roles of elements • and ∘ are redundant, as they point to the same components, *σ*_☐_ and *ρ*_♠_. For instance, the one-element wedge (*D*, *ω*) does not factor through *D*″ uniquely, i.e. there exist two maps *u*, *u*′: *D* → *D*″ such that *ω* = *ω*″ ∘ *u* and *ω* = *ω*″ ∘ *u*′. Satisfying both existence and uniqueness conditions to be an end requires a three-element wedge. (NB. The empty set also constitutes a wedge—naturality is trivially satisfied, since composition with an empty function is an empty function.).

To summarize, ends obtain as optimal constraint satisfaction, where the constraints are implicitly specified by feedback on stimulus-response trials for the tasks. Sets Hom(*S*, *S*) and Hom(*R*, *R*) consist of the possible maps between trigrams within a task, and Hom(*S*, *R*) consists of the possible maps of trigrams between tasks. Commutativity constrains the candidate solution sets to only those sets whose elements pick out the trigram mappings for each task instance that conjointly satisfy equivariance between task instances. Universality further constrains the candidates to only those sets whose elements are necessary *and* sufficient for commutativity, i.e. the relational schema (monoid) common to all task instances. In this way, relational schema induction is a form of optimal constraint satisfaction.

#### Schema computation

Computing an end is basically a “directed” search over a collection of wedges. A wedge consists of a family of morphisms picking out actions on trigrams. For example, let (*S*, *σ*) and (*R*, *ρ*) represent tasks *τ*_1_ and *τ*_2_ (Fig 1), respectively. So, *σ*_△_, *σ*_☐_, *σ*_♡_ ∈ Hom(*S*, *S*) and *ρ*_♣_, *ρ*_♠_, *ρ*_★_ ∈ Hom(*R*, *R*). We fix an equivariant map *f*_0_: BEH ↦ HUQ, FUT ↦ KES, PEJ ↦ NIZ. (There are six possible equivariant maps from *S* to *R*, since the maps are relabeled permutations of *S*.) Inducing an action (component) amounts to supposing a wedge consisting of an element and a map for each hom-set. Let that element be indicated as 0, since their identifiers are unimportant beyond being distinguishable. Suppose we pick the no-rotation action for the first task, i.e. *ω*_*S*_: 0 ↦ *σ*_△_. The dinaturality condition requires the corresponding action in the second task, which is *ρ*_♣_, i.e. *ω*_*R*_: 0 ↦ *ρ*_♣_. Selection proceeds as a form of “hypothesis generation and test” until the dinaturality condition is satisfied. For instance, suppose we hypothesized *ρ*_♠_ as the other action. The commutativity test fails, i.e. *f*_0_(*σ*_△_ (BEH)) = HUQ ≠ KES = *ρ*_♠_(*f*_0_(BEH)), so this hypothesis is rejected and a new action is selected. Thus, we have the wedge (*D*, *ω*), where *D* is the set {0} and *ω* is the pair of maps pointing to (*σ*_△_, *ρ*_♣_). However, this wedge is not an end, because there exist two other pairs of mappings that satisfy the commutativity condition. We require a larger set that includes *D*. We repeat the process by adding another element and a pair of mappings to satisfy commutativity: e.g., the wedge (*D*′, *ω*′), where *D*′ = {0, 1} and *ω*′ includes the mappings 1 ↦ *σ*_☐_ and 1 ↦ *ρ*_♠_. As sets, we have the order (inclusion) relation *D* → *D*′, which is used as the basis for directed search to the end.

The equivariant maps used to determine the end are not given by stimulus-response feedback, since they are maps between tasks. However, these maps are inferred from the component form of the equivariance condition (definition 11), i.e. *f*(*σ*_*a*_(*s*)) = *ρ*_*a*_(*f*(*s*)), as *σ*_*a*_ and *ρ*_*a*_ are implicitly given by feedback on the within-task trigram responses. Note that the component actions are indexed by the elements of the monoid, *a* ∈ *M*. From the participant’s perspective, however, these components are indexed by shapes, which change from task to task. So, participants must also determine the correspondence between shapes in a “hypothesis generation and test” manner, as mention above: e.g., hypothesize that △ corresponds to ♣ and test that the map *f* satisfies equivariance, i.e. *f*(*σ*_△_(*s*)) = *ρ*_♣_(*f*(*s*)). If no correspondence between shapes satisfies equivariance, then *f* is also rejected.

**Fig 5:**
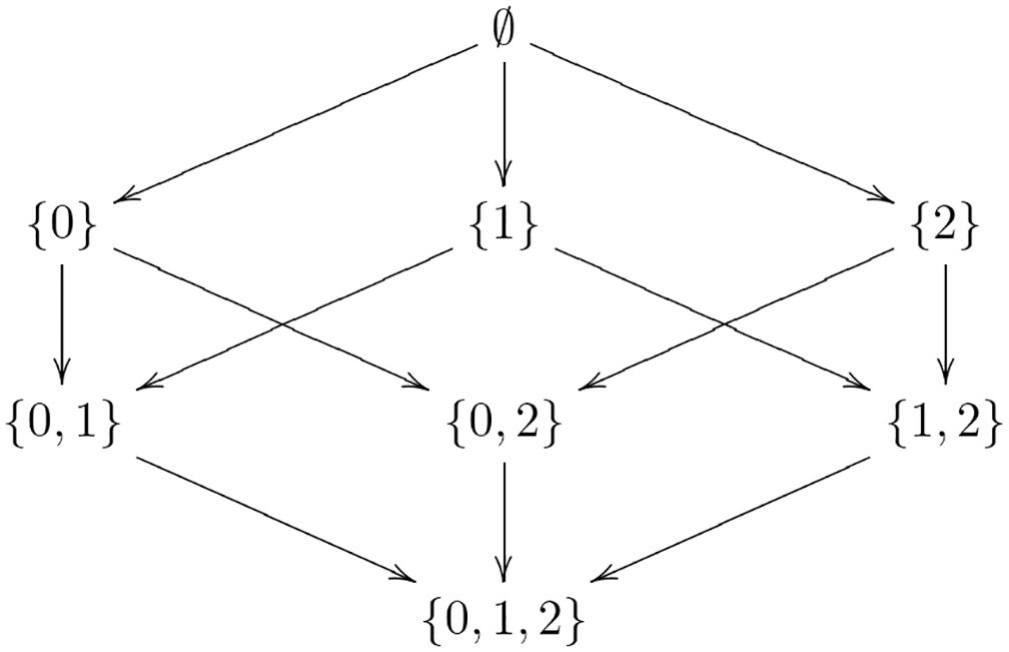
The preordered set of wedges with the end as the set {0, 1, 2}.

With regard to the existence condition, there is a (sub)collection of wedges forming a *preordered set* (definition 14), where the end is the *terminal object* (definition 53). The objects are sets whose elements correspond to the elements of the monoid from which the tasks were constructed. The sets are ordered by inclusion (Fig 5). Thus, computing the end is a matter of following the arrows to the terminal object, which corresponds to the monoid. Clearly, from any starting point—object in the preordered set—one obtains the end by arrow traversal. The beginning of the induction paradigm corresponds to the empty set, which is also the *initial object* (definition 55). The psychological interpretation is having no *a priori* knowledge of the actions.

Note that although the existence condition can be satisfied with sets containing more than three elements, sets with more than three elements fail to satisfy the uniqueness condition. For example, a wedge consisting of set *D*″ = {0, 1, 2, 3} satisfies existence, but not uniqueness, because at least one element is redundant as there are only three unique action components. Suppose elements 2 and 3 map to the same pair (*σ*_♡_, *ρ*_★_). Element 3 (2) is sufficient, but not necessary, as commutativity is also achieved by element 2 (3). Hence, the sets are also ordered by containment with regard to the uniqueness condition, *D*″ → *D*′. The intersection of the collection of sets satisfying the existence condition and the collection of sets satisfying the uniqueness condition is the end, consisting of the set *E* = {0, 1, 2} together with the pairs of maps satisfying commutativity.

Note that any 3-element set together with the associated family of morphisms is an end for this situation. However, as already mentioned, such alternatives are essentially the same with regard to the universal mapping property for ends.

#### Transfer as completion

Having induced the monoid, *M*, learning transfer obtains from a “completion” process. For example, suppose a new set of shapes, *Sh*′ = {⊕, ♢, ⊡} and trigrams, *Tri*′ = {DOH, MUV, RIY}. On seeing that (⊕, DOH) maps to DOH one infers that ⊕ corresponds to the unit (or, 0° rotation) action; hence, correctly predicts that (⊕, MUV) maps to MUV and (⊕, RIY) maps to RIY. Similarly, on seeing that (♢, DOH) maps to MUV one infers that ♢ corresponds to a non-zero rotation; hence, correctly predicts (♢, MUV) maps to RIY and (♢, RIY) maps to DOH. Note that one (non-zero) rotation can be used to infer (by completion) the other rotation: e.g., ♢ ⋅ ♢ = ⊡, as all elements of a monoid (as morphisms of a one-object category) are compatible morphisms implying their composite (see definition 1).

As already mentioned (Introduction) the number of first trial errors will depend on the distribution of cues. Assuming different shape cues and no forgetting of feedback, two information trials are necessary and sufficient for correct prediction of targets for the other seven cues.

Completion is also a universal construction, i.e. specifically, the *free M-set representation* for the given set (definition 70). This construction is functorial (remark 71), and is the *left adjoint* (definition 72)—“pseudo-inverse” (remark 73)—to the forgetful functor (example 74). Each set *S* is sent to the M-set representation (*M* × *S*, *μ* × 1_*S*_), where *μ* is the multiplication for the monoid *M*. The free M-set pairs every element in *S* with every pairwise combination of actions, i.e. *μ* × 1_*S*_: ((*a*, *b*), *s*) ↦ (*a* ⋅ *b*, *s*). The table of actions is completed from combinations of actions inferred from the information trials. For example (above), knowing (♢, DOH) ↦ MUV yields (⊡, DOH) ↦ RIY as the free M-set action *μ* × 1_S_((♢, ♢), DOH), i.e. the action of ♢ on DOH yields MUV, and the action of ♢ on MUV yields RIY. In this way, transfer is afforded by a universal completion process.

### Other relational schemas

**Fig 6:**
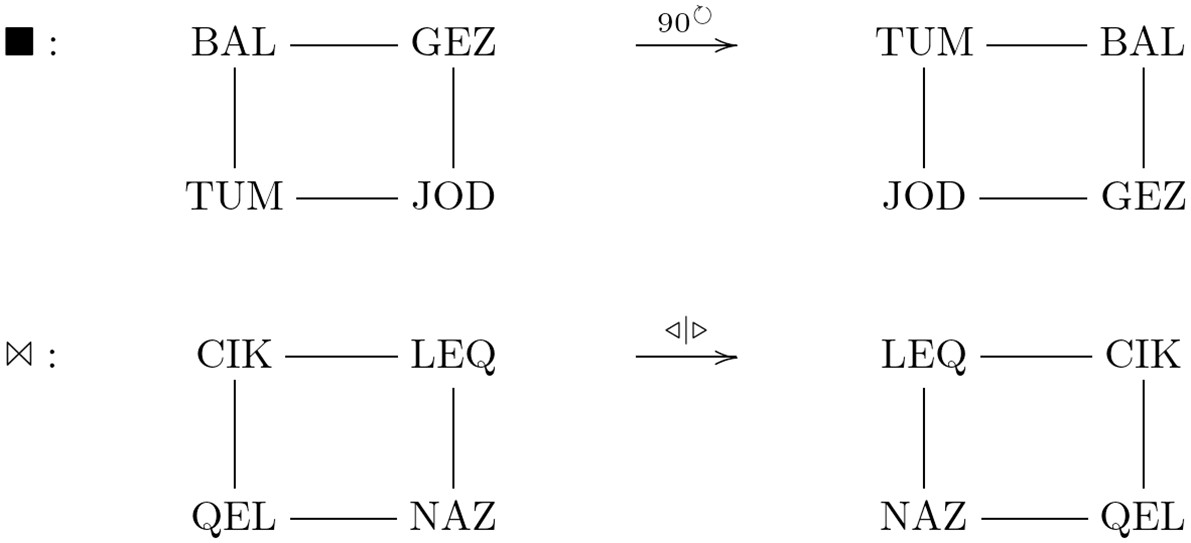
Examples of cyclic and reciprocal forms of relational schema induction.

The reconstruction theory is general and applies to other examples of relational schema induction [9, 10], which we consider here. The relational schema induction paradigm essentially proceeds as described in the previous sections. However, for the examples presented here, only some actions (shapes) of a monoid specify the learning tasks. Specifically, participants were examined on two other group-like structures, where the actions (shapes) constitute partial *cyclic-4* and *Klein-4* groups [9], as exemplified in Fig 6. In the cyclic-4 case, one shape corresponds to a 90 clockwise rotation, e.g., (◼, BAL) maps to TUM, (◼, GEZ) maps to BAL, etc. (Fig 6, top row), and the other shape corresponds to a 90° anticlockwise rotation, e.g., (●, BAL) maps to GEZ, etc. In the Klein-4 case, one shape corresponds to horizontal reflection, e.g., (⋈, CIK) maps to LEQ and (⋈, LEQ) maps to CIK, etc. (Fig 6, bottom row), and the other shape to vertical reflection, e.g., (☼, CIK) maps to QEL and (☼, QEL) maps to CIK, etc.

The mean number of first-trial errors on the fourth task for the cyclic and Klein group-like cases were 2.67 and 2.00, respectively [9], experiment 1. These data accord with the two information trials needed to determine the targets for the remaining six cues. Transfer proceeds as with the cyclic-3 example. Assuming distinct shape cues and no forgetting, two information trials are necessary and sufficient to predict the targets for the other six cues.

Although these two examples involve only two of the four components that constitute the monoid (group) actions, the other components are automatically given by the composition operation for categories. Recall that for every pair of compatible morphisms, *f*: *A* → *B* and *g*: *B* → *C* there is a composite morphism *g* ∘ *f*: *A* → *C*. The components of a monoid action are endofunctions (i.e., map to/from the same set), hence every pair of components is a pair of compatible morphisms implying their composites. So, for example (cyclic-4), given that (◼, (BAL) maps to TUM, (◼, GEZ) maps to BAL, we have the composite (◼ ∘ ◼, BAL) maps to JOD, etc. A similar situation arises with the Klein-4 example. For instance, (⋈, CIK) maps to LEQ and (☼, LEQ) maps to NAZ, hence their composite (☼ ∘ ⋈, CIK) maps to NAZ, which corresponds to diagonal reflection. Thus, the monoids are induced. However, for new task instances, the assignment of the other two shapes to the other two actions is ambiguous: a third information trial is needed to determine which of the remaining two shapes corresponds to which of the remaining two actions.

A later study [10] employed three components of the cycle-6 group: no-rotation, 60° clockwise rotation, and 60° anticlockwise rotation. The reconstruction theory applies in the same way as for the the previous examples. The difference is that more information trials are needed to determine the order of stimuli for the new task.

## Discussion

We return to the broader questions about the implications of learning transfer for the nature of cognition, raised earlier, in the light of our reconstruction theory of relational schema induction.

### Relations versus associations

The relational schema induction paradigm was introduced to assess whether learning transfer depends on relational or associative processes [9]. On one hand, some authors have argued that developmental differences depend on a capacity to process relational information [7, 8]. However, other authors argued that associative processes are sufficient [5, 19]. Our approach shows how these disparate views may be reconciled.

To induce the common structure, subjects must first learn the basic cue-target relations. These relations are task-specific, so can also be regarded as a (learned) set of stimulus-specific associations. The trigram-trigram associations for a task *S* and a given action constitute a map in the set Hom(*S*, *S*). Thus, there is an associative (first-order) component to induction. However, to recover the structure, subjects must also compute an end by comparing the task-specific relations (associations) for consistency across tasks via the equivariant maps. The elements constituting the end are independent of specific task stimuli. So, there is also a task-independent, relational (second-order) aspect to schema induction. These associative and relational aspects can be independently manipulated as the cardinalities of set *S*: number of elements acted on, and monoid *M*: number of components acting on *S*, respectively. Hence, the empirical implications of associative and relational information on learning transfer can both be assessed.

### Quality versus quantity

An important implication of our theory, which can be tested empirically, pertains to qualitative versus quantitative differences in learning transfer with regard to configural associative learning tasks. Configural association involves responses that depend on pairs of cues. The shape-colour example (Introduction) is an instance of a configural association. Learning configural associations can take many repeated learning trials, even for adult humans [20]. However, configural association can also be seen as a kind of relational schema [9], in which case, our theory predicts a form of learning transfer from a single information trial when configural associations are regarded as relational schemas.

Suppose participants are required learn to associate cues to targets depending on context: e.g., in the context of a green display background, triangle is associated to triangle and square is associated to square; in the context of a blue background, triangle associates to square and square associates to triangle. After learning these associations a new instance of configural association is administered. This new instance consists of different shapes and colours: e.g., in the context of a yellow display background, circle associates to circle and cross associates to cross; in the context of a brown background, circle associates to cross and cross associates to circle. This situation can be regarded as a monoid action on a set: e.g., whereby colours correspond to actions on shapes, such as the map G: △ ↦ △, G: ☐ ↦ ☐, B: △ ↦ ☐, B: ☐ ↦ △. In this case, the monoid corresponds to the cyclic-2 group, Z/2Z. Accordingly, the collection of such tasks and their equivariant maps forms a category, and the monoid is reconstructed by computing the end of the functor Hom(*U*−, *U*−), as in the previous examples. The monoid is then applied to a new task instance given a single information trial, affording target prediction for the other three cues. The empirically testable implication is that a new configural association can be acquired following a single information trial, in contrast to the multi-shot, repetitive learning typically seen in associative learning paradigms [20].

As mentioned in the Introduction, an associative learning model could also be developed to demonstrate learning transfer in this situation, by varying the learning rate with tasks. Indeed, a popular early method for accelerating learning was to include a “momentum” term in backpropagation-style neural network learning models [21], which modulates the learning step for faster learning. In this way, the number of trials to criterion may decrease with tasks, thereby demonstrating transfer. However, the associative learning model still requires training on all four cue-target pairs. The broader point, here, is that the (overly simple) notion of learning transfer as “accelerated” learning belies qualitative differences in the underlying mechanism.

### Humans versus non-humans

Qualitative differences in learning transfer also have implications for comparative psychology. As mentioned earlier, a capacity to process relational information is often seen as a characteristic of cognition best demonstrated, if not uniquely so, in humans [7, 8, 18]. An often used method for demonstrating relational processing in non-humans is the *relational match to sample* paradigm, whereby subjects are trained to respond on the basis of the sameness/difference relation. The crucial test is first-trial errors for novel stimuli. Bees, for example, show significantly better than chance performance on novel stimulus pairs [22], suggesting that a capacity for abstract relations is not unique to humans, and indeed may be wide spread among other species. Learning set transfer tasks as applied to non-humans can be viewed in terms of relational schema induction and thereby used as a basis for comparison and contrast [18]. In this way, our reconstruction theory has implications for learning transfer in other species.

For instance, the structure of sameness/difference is also isomorphic to the cyclic-2 group: the relations same(A, A), same(B, B), different(A, B), different(B, A) can be interpreted as actions on sets. Let *s* and *d* denote sameness and difference, respectively. As actions on sets we have *s*: *A* ↦ *A*, *B* ↦ *B* and *d*: *A* ↦ *B*, *B* ↦ *A*. However, unlike the induction paradigms, the relation (action) is not indicated by a specific (unique) stimulus that changes with each instance of the task, which affords learning transfer without any information trials. By contrast, for relational schema induction, one information trial is required to determine whether a stimulus (shape) corresponds to the no-rotation, or rotation action. For comparison with the cyclic-3 task, suppose the same shapes are used for the same actions in each learning task. In this situation, induction reduces to identifying the order of the novel trigrams, which requires just one (non-zero rotation) information trial.

Clearly, a capacity to represent relations independent of specific stimuli is a necessary step. However, the relational schema induction paradigm highlights how humans also have the capacity to apply those relations to generate a response—*generativity test* [10]. Our reconstruction theory helps clarify this distinction in that the end has both the elements *and* the structure of such actions, i.e. the monoid underlying each task. The relational match to sample paradigm only requires a response dependent on the identity of a relation, it does not require the subject to generate a response given a relation as an action on a given stimulus. What is unclear is whether (or, to what extent) non-humans have this ability. From our perspective, relational schema induction necessitates the extra step of inducing the associated algebraic structure. A benefit of our reconstruction theory is to bring such comparisons/contrasts between cohorts into sharper relief.

### Cognitive complexity

An earlier version of the relational schema induction paradigm was used to reveal developmental differences in the capacity to demonstrate transfer [23]. Younger children had greater difficulty at learning transfer than older children when the number of actions was increased. The authors also used a category theory approach to model this difference, in terms of computing commutativity, although they did not address the induction aspect of the task, which we have done here. Their essential point was that younger children are more limited in the number of stimulus relations (component actions) that they could compare for assessing the correspondence between tasks as the basis for transfer. Subsequent empirical work over a wide variety of tasks yielding analogous differences led to the *relational complexity theory* of cognitive capacity [8, 18]. A task that requires assessing a relationship between three stimuli (ternary relation) is generally more difficult than a task requiring at most two (binary relation). A binary (ternary) relation is a subset of a binary (ternary) product of sets. Each set may be regarded as a “dimension of (task) variation” whence a measure of task difficulty (cognitive complexity) is the number of task dimensions [8, 18].

Our reconstruction theory is compatible with relational complexity theory as the set *E*, constituting the end, is isomorphic to the underlying monoid. Each element of *E* identifies with a distinct action (component) on a set of trigrams, which can be regarded as a dimension of task variation. So, for example, induction and transfer for tasks generated from the cyclic-3 group should be more difficult than for tasks generated from the cyclic-2 group. Note that difficulty is not simply a matter of increased memorization demands, as a memorization strategy does not afford the same level of learning transfer [10].

Categorical products provide a formal connection to relational complexity. Ends can be succinctly defined in terms of products [14], and products were used to provide a category theory treatment of cognitive complexity [24]. However, for ends, the products involve all objects in the category. This situation suggests that cognitive complexity also increases with the number of task instances (objects in the category of tasks), which is clearly not the case as learning becomes easier as one progresses through the series of learning tasks. A more psychologically plausible explanation is that an *n*-ary product is constructed from binary products, by natural isomorphism: e.g., *A* × *B* × *C* ≅ (*A* × *B*) × *C*. Such situations correspond to a *segmentation* strategy, whereby excessive cognitive complexity is supposed to be circumvented by a serial process involving lower arity relations at each step [8, 18].

### Structure mapping

Notice that although the relational schema induction and learning set paradigms (as considered here) involve one-to-one correspondence between the elements (e.g., shapes and trigrams) of different task instances, this principle is derived by our approach, not assumed. Relational schema induction was considered to involve the mapping of structure [9], for example, as specified by *structure mapping theory* [11]. However, structure mapping theory and related models of analogy generally assume one-to-one correspondence (isomorphism), as a hard, or soft constraint on mapping [12]. By contrast, the equivariant maps used to compute ends need not be isomorphisms. So, reconstruction theory implies a further generalization of the induction paradigm whereby tasks are homomorphically, but not just isomorphically related. This situation arises when the sets acted on have different numbers of elements. For instance, suppose in one task the set acted on consists of three stimuli, *S* = {*a*, *b*, *c*}, and another task consists of four stimuli *R* = {*x*, *y*, *z*, *w*} for the cyclic-3 action. The action on two elements in *R* is the same, say *z* and *w*. Yet, the monoid is still recoverable in these situations, which affords an empirically testable prediction for the corresponding capacity in humans.

### Systematicity of inference

The systematic consistency with which subjects induce the relational schema and transfer this knowledge across task instances [9, 10] raises a familiar systematicity challenge [15]. In the current context, this challenge is to explain *why* subjects who exhibit transfer on one task also exhibit transfer on another task. This property pertains to a systematicity of learning, or *second-order systematicity* [25, 26].

A category theory explanation is that systematicity obtains from a universal construction [16]. Ends are universal constructions [14]. Hence, the systematicity property suggested by the relational schema induction experiments [9, 10] follows from computing the end of the appropriate functor. Computing the end (or, universal morphism) follows from another kind of universal process: *categorical (co)recursion* [27]. In a formal sense, “All roads lead to Rome” as shown by example (Schema computation). The process terminates because the end is the terminal object, pointing to itself. So, the current work affords both structural and computational explanations for relational schema induction.

The same form of explanation for systematicity also applies to the learning transfer aspect of relational schema induction, as learning transfer also involves a universal construction, i.e. the free M-set construction, which is left adjoint to the forgetful functor. The *general adjoint functor theorem* (see [28], theorem 6.3.10) implies that a forgetful functor from any category of algebras has a left adjoint (see [28], p. 163). Another example of a free-forgetful adjunction was used to explain the systematicity of transfer aspect of relational schema induction for the Klein-4 schema [16].

### Further work

New approaches raise new questions and directions for further work. In this paper, we focused on one-shot transfer, because it is regarded as a hallmark of human-level transfer [18]. An important direction for further work is to extend this approach to the probabilistic setting. Further work is needed to understand the link between one-shot and multi-shot learning transfer, as commonly exhibited in non-human studies [1, 2].

Our approach has been to consider a more general theory to incorporate apparently different forms of cognitive process: relational versus associative learning. Yet, more general theory seems more removed from the underlying neuroscience, which raises questions about the link to the neurocomputational system. As observed elsewhere [29], some category theory constructions pertaining to constraint satisfaction, like the ones employed here, are reminiscent of a neural network model of analogy [30]. So, another direction is to investigate the formal links between the current theory and such models.

Our reconstruction theory does not say why subjects can fail to induce the relevant structure. Relational complexity theory attributes such failures to differences in working memory capacity limits for different cohorts [8, 18]. One possible way of addressing such differences is to incorporate a category theory approach to resources [31] for application to relational schema induction. In such situations, *enriched category theory* can be applied, where the hom-sets have additional structure—beyond being just sets—to model the implementational aspects of a task [32, 33].

An enriched setting could be used to explicitly model learning in response to error feedback. To illustrate, suppose the task is to learn a mapping from a set *A* to a set *B*. The function space, Hom(*A*, *B*), can be enriched with an ordering in terms of the number of errors relative to the target function to be learned: *f* ≤_*t*_ *g*, meaning that function *f* yields fewer errors than function *g* with regard to target function *t*. In this situation, Hom(*A*, *B*) together with the preorder ≤_*t*_ constitutes a preordered set, hence a category, with *t* as the initial object, since *t* ≤_*t*_ *f* for all *f* in Hom(*A*, *B*). Thus, learning an individual task (target map) also involves a universal construction, i.e. constructing the initial object.

The current work considers relational schema induction for learning tasks where the underlying action is a monoid, hence a one-object category. However, the theory generalizes to other algebraic structures as categories with more than one object (theorem 68). We expect that this more general theory will enable us to address induction problems more broadly, in future work.

## Methods

For deeper introductions to the category theory presented here, see [14, 28]. For pedagogical introductions, including Tannakian reconstruction as an end, see [34].

### Categories, functors and natural transformations

**Definition 1** (Category). A *category* **C** consists of a collection of *objects*, O(C)={A,B,…}, a collection of *morphisms*, M(C)={f,g,…}—a morphism written in full as *f*: *A* → *B* indicates object *A* as the *domain* and object *B* as the *codomain* of *f*—including for each object A∈O(C) the *identity morphism* 1_*A*_: *A* → *A*, and a *composition* operation, ∘, that sends each pair of *compatible* morphisms *f*: *A* → *B* and *g*: *B* → *C* (i.e. the codomain of *f* is the domain of *g*) to the *composite* morphism *g* ∘ *f*: *A* → *C*, that together satisfy the laws of:

- *identity*: *f* ∘ 1_*A*_ = *f* = 1_*B*_ ∘ *f* for every morphism f∈M(C), and
- *associativity*: *h* ∘ (*g* ∘ *f*) = (*h* ∘ *g*) ∘ *f* for every triple of compatible morphisms f,g,h∈M(C).

Composition of *f*, *g* and *h* is also written *h* ∘ *g* ∘ *f*, since ∘ is associative, and the operation is also denoted ∘_**C**_ to make the category explicit.

**Remark 2**. The collection of morphisms in **C** with domain *A* and codomain *B* is called a *hom-set*, denoted Hom_**C**_(*A*, *B*), or Hom(*A*, *B*) when the category is clear.

**Example 3** (**Set**). The collection of sets and functions between sets forms a category, denoted **Set**. The identity morphisms are the identity functions. Composition is composition of functions. Hom_**Set**_(*A*, *B*) is the set of functions from set *A* to set *B*, which is also called the *function space* and denoted *B*^*A*^.

**Definition 4** (Opposite category). The *opposite category* to a category **C** is the category, denoted **C**^op^, that has:

- the objects of **C**,
- the “reversed” morphisms of **C**, i.e. a morphism *f*: *A* → *B* in **C** is the morphism *f*^op^: *B* → *A* in **C**^op^, and
- “swapped” composition of **C**, i.e. if *f* ∘ *g* is a composite in **C**, then *g*^op^ ∘ *f*^op^ is a composite in **C**^op^.

A morphism *f*^op^ is also simply denoted *f* when the category is understood.

**Definition 5** (Monoid). A *monoid* (*M*, ⋅, *e*) consists of a set *M*, a (closed) binary operation ⋅, called *multiplication*, and an element *e* ∈ *M*, called the *unit*, such that multiplication is:

- *unital*: *a* ⋅ *e* = *a* = *e* ⋅ *a* for every element *a* ∈ *M*, and
- *associative*: *a* ⋅ (*b* ⋅ *c*) = (*a* ⋅ *b*) ⋅ *c* for every triple of elements *a*, *b*, *c* ∈ *M*.

A monoid is also simply denoted by its underlying set, *M*.

**Example 6** (Integers). The integers constitute monoids.

1. (Z,+,0): the integers together with addition.
2. (Z,×,1): the integers together with multiplication.
3. Z/2Z: {0, 1} together with addition modulo-2.
4. Z/nZ: {0, …, *n*−1} together with addition modulo-*n* for *n* > 0.

**Remark 7**. Z/nZ is also a *group*, i.e. a monoid where every element *a* has an inverse: an element *b* ∈ *M* such that *a* ⋅ *b* = *e* = *b* ⋅ *a*. Z/nZ is called a *cyclic group*.

**Remark 8**. A monoid (*M*, ⋅, *e*) is a one-object category: each element *a* ∈ *M* is the morphism *a*: * → *, with the unit as the identity morphism, and composition given by the monoid operation, i.e. *b* ∘ *a* corresponds to *a* ⋅ *b*. Accordingly, Hom_*M*_(*, *) = *M*. Moreover, as the arguments to the composition operation are swapped, the monoid is an opposite category, denoted *M*^op^, i.e. we have $a\circ_{M^{\text{op}}}b=b\circ_{M}a=a\cdot b$. Hence, ${\text{Hom}}_{M^{\text{op}}}(*,*)$ has the structure of the monoid *M*.

**Proposition 9** (Monoid). The hom-set ${\text{Hom}}_{M^{\text{op}}}(*,*)$ has the structure of monoid *M*, i.e. ${\text{(Hom}}_{M^{\text{op}}}(*,*),\circ ,e)=(M,\cdot ,e)$.

*Proof*. See remark 8.

**Definition 10** (Monoid action, M-set). Let (*M*, ⋅, *e*) be a monoid, and *S* a set. A *(left) monoid action* on *S* is a function *σ*: *M* × *S* → *S* that satisfies:

- *identity*: *σ*(*e*, *s*) = *s*, and
- *compatibility*: *σ*(*a* ⋅ *b*, *s*) = *σ*(*a*, *σ*(*b*, *s*))

for every *a* ∈ *M* and *s* ∈ *S*. The set *S* is called an *M-set*, and the pair (*S*, *σ*) is called an *M-set representation*. The action *σ* is also called an *M-act*. M-set representation (*M*, *μ*) is also denoted *M*^*l*^, representing the action of *M* on itself.

**Definition 11** (Equivariant map). Let (*M*, ⋅, *e*) be a monoid, and (*S*, *σ*) and (*R*, *ρ*) M-set representations for *M*. An *equivariant map* is a function *f*: *S* → *R* that is compatible with the actions: *f*(*σ*(*a*, *s*)) = *ρ*(*a*, *f*(*s*)) for all *a* ∈ *M* and *s* ∈ *S*.

**Remark 12**. The collections of M-set representations and equivariant maps constitute a category, denoted **MSet**.

**Definition 13** (Isomorphism). A morphism *f*: *A* → *B* in **C** is called an *isomorphism* if there exists a morphism *g*: *B* → *A* in **C** such *f* ∘ *g* = 1_*B*_ and *g* ∘ *f* = 1_*A*_. Morphism *g* is called the *inverse* of *f* and *B* is said to be *isomorphic* to *A*, written *A* ≅ *B*.

**Definition 14** (Preordered set). A *preordered set* (*P*, ≤) is a set *P* with an order relation ≤ that is *reflexive* (i.e. *p* ≤ *p* for all *p* ∈ *P*) and *transitive* (i.e. *p* ≤ *q* and *q* ≤ *r* implies *p* ≤ *r* for all *p*, *q*, *r* ∈ *P*).

**Remark 15**. A preordered set, (*P*, ≤), is a category whose objects are the elements *p* ∈ *P* and morphisms are the order relations, i.e. there is a morphism *p* → *q* whenever *p* ≤ *q*. The identities are given by reflexivity and composition by transitivity.

**Definition 16** (Product). In a category **C**, a *product* of objects *A* and *B* is an object *P* together with a pair of morphisms $\overset{´}{\pi }:P\to A$ and $\overset{`}{\pi }:P\to B$, called *projections*, such that for every object *Z* and pair of morphisms *ϕ*: *Z* → *A* and *ψ*: *Z* → *B* there exists a unique morphism *u*: *Z* → *P* such that $f=\overset{´}{\pi }\circ u$ and $g=\overset{`}{\pi }\circ u$. The canonical product for *P* is denoted *A* × *B*, and *u* is denoted 〈*ϕ*, *ψ*〉 as it is determined by *ϕ* and *ψ*.

**Example 17** (Cartesian product). In **Set**, the product of sets *A* and *B* is the *Cartesian product*: *A* × *B* = {(*a*, *b*)|*a* ∈ *A*, *b* ∈ *B*} and projections $\overset{´}{\pi }:(a,b)\mapsto a$ and $\overset{`}{\pi }:(a,b)\mapsto b$. The unique morphism, *u*, is the function 〈*ϕ*, *ψ*〉: *z* ↦ (*ϕ*(*z*), *ψ*(*z*)).

**Definition 18** (Functor). A *functor* is a “structure-preserving” map from a category **C** to a category **D**, written *F*: **C** → **D**, sending each object *A* and morphism *f*: *A* → *B* in **C** to the object *F*(*A*) and the morphism *F*(*f*): *F*(*A*) → *F*(*B*) in **D** (respectively) that satisfies the laws of:

- *identity*: *F*(1_*A*_) = 1_*F*(*A*)_ for every object A∈O(C), and
- *compositionality*: *F*(*g* ∘_**C**_ *f*) = *F*(*g*) ∘_**D**_ *F*(*f*) for every pair of compatible morphisms f,g∈M(C).

**Example 19** (Identity functor). The *identity functor* for a category **C**, written 1_**C**_: **C** → **C**, sends every object/morphism to itself, i.e. 1_**C**_: *A* ↦ *A*, *f* ↦ *f*.

**Example 20** (Diagonal functor). The *diagonal functor*, written Δ: **C** → **C** × **C**, sends every object/morphism to the pair of itself, i.e. Δ: *A* ↦ (*A*, *A*), *f* ↦ (*f*, *f*).

**Example 21** (Product functor). The *product functor*, written Π: **C** × **C** → **C**, sends every pair of objects/morphisms to their products, i.e. Π: (*A*, *B*) ↦ *A* × *B*, (*f*, *g*) ↦ *f* × *g*.

**Example 22** (Underlying/forgetful functor). The *underlying functor* *U*: **MSet** → **Set** sends each M-set (*S*, *σ*) to its underlying set *S*. This functor is also called a *forgetful functor*, as it forgets the actions.

**Remark 23**. A functor *F*: **C**^op^ → **D** is called a *contravariant functor*; a functor *F*: **C** → **D** is called a *covariant functor*.

**Definition 24** (Transpose, component). Let (*M*, ⋅, *e*) be a monoid, *S* a set, and *σ*: *M* × *S* → *S* an M-act on *S*. The *transpose* of *σ*, denoted $\tilde{\sigma }$, is the map $\tilde{\sigma }:M\to (S\to S);a\mapsto (\sigma_{a}:s\mapsto \sigma (a,s))$. The map *σ*_*a*_ is called a *component* of *σ*.

**Remark 25**. The transpose of *σ* is the (contravariant) functor $\tilde{\sigma }:M^{op}\to \text{Set}$ that sends each morphism *a*: * → * to the component *σ*_*a*_: *S* → *S*.

**Definition 26** (Image). Let *F*: **C** → **D** be a functor. The *image* of *F* is the collection of objects {F(A)|A∈O(C)} and morphisms {F(f)|f∈M(C)}.

**Remark 27**. A *commutative diagram* expresses a collection of equational relations between pairs of morphisms: any two paths of arrows beginning at the same object and finishing at the same object, where one path consists of at least two (non-identity) arrows. Such diagrams are sometimes referenced by their shape.

**Definition 28** (Natural transformation). Let *F*, *G*: **C** → **D** be functors. A *natural transformation*, written $\eta :F\dot{\to }G$, is a family of **D**-morphisms $\left\{\eta_{A}:F(A)\to G(A)|A\in O(C)\right\}$ such that *G*(*f*) ∘ *η*_*A*_ = *η*_*B*_ ∘ *F*(*f*) for every morphism *f*: *A* → *B* in **C**, as indicated by the following commutative square:

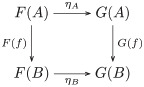
(1)

**Example 29** (Projections). Suppose projection functors $\overset{´}{\Pi }:(A,B)\mapsto A$ and $\overset{`}{\Pi }:(A,B)\mapsto B$. The product and projection functors are related by natural transformations, i.e. $\overset{´}{\pi }:\Pi \dot{\to }\overset{´}{\Pi }$ and $\overset{`}{\pi }:\Pi \dot{\to }\overset{`}{\Pi }$, as indicated by the following two commutative diagrams:

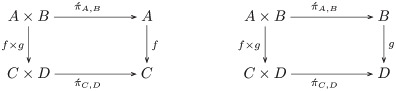
(2)

**Definition 30** (Natural isomorphism). A *natural isomorphism*, written *η*: *F* ≅ *G*, is a natural transformation such that each component *η*_*A*_: *F*(*A*) → *G*(*A*) is an isomorphism.

**Example 31** (Passive product). The arguments to a product are swapped (i.e, put in passive form) by the natural isomorphism $\overset{`}{\pi }\times \overset{´}{\pi }:A\times B≅B\times A$, as indicated by the following commutative diagram:

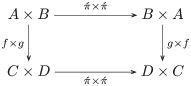
(3)
Compare *John loves Mary* (active) and *Mary is loved by John* (passive).

**Remark 32**. Let $\tilde{\sigma },\tilde{\rho }:M\to \text{Set}$ be functors, i.e. the transposes of M-acts *σ* and *ρ* on sets *S* and *R*, respectively. The equivariant map *f*: *S* → *R* is the natural transformation $f:\tilde{\sigma }\dot{\to }\tilde{\rho }$, as indicated by the following commutative diagram:

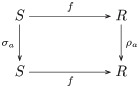
(4)
which says that *f* ∘ *σ*_*a*_ = *ρ*_*a*_ ∘ *f* for each element *a* ∈ *M* and *s* ∈ *S*.

**Definition 33** (Hom-operations). The following *hom-operations* are defined.

1. f_*_: *g* ↦ *f* ∘ *g*.
2. f*: *g* ↦ *g* ∘ *f*.
3. f* g_*_: *h* ↦ *g* ∘ *h* ∘ *f*.

**Remark 34**. These operations provide a convenient notation for defining the following functors pertaining to hom-sets.

**Definition 35** (Hom-functors). The following *hom-functors* are defined.

1. Hom_**C**_(*A*, −): **C** → **Set**; *X* ↦ Hom(*A*, *X*), *f* ↦ f_*_.
2. ${\text{Hom}}_{C^{\text{op}}}(-,A):{\text{C}}^{\text{op}}\mapsto \text{Set};X\mapsto \text{Hom}(X,A),f\mapsto f*$ .
3. ${\text{Hom}}_{C^{\text{op}}\times \text{C}}(-,-):(A,B)\mapsto \text{Hom}(A,B),(f,g)\mapsto f*g_{*}$.

**Example 36** (Multiplication). The multiplication operation of a monoid, *μ*: *M* × *M* → *M* corresponds to the hom-functor ${\text{Hom}}_{M^{\text{op}}}(*,-):*\mapsto M,a\mapsto \mu_{a}$.

**Remark 37**. Hom-functors can be (pre)composed with other functors. Suppose functors *F*: **C** → **D** and *G*: **D**^op^ → **C**^op^. We have:

1. ${\text{Hom}}_{{\text{D}}^{\text{op}}\times \text{D}}(-,F-):(g,f)\mapsto (g^{*}Ff_{*}:h\mapsto Ff\circ h\circ g)$, and
2. ${\text{Hom}}_{{\text{C}}^{\text{op}}\times \text{C}}(G-,-):(g,f)\mapsto (Gg^{*}f_{*}:h\mapsto f\circ h\circ Gg)$.

Simplifying, we write Hom_**D**_(−, *F*−) and Hom_**C**_(*G*−, −).

**Remark 38**. Hom-functors are related by natural (hom-)transformations. For a morphism *h*: *A* → *B* in **C** the following natural transformations are defined.

1. $\text{Hom}(h,-):\text{Hom}(A,-)\dot{\to }\text{Hom}(B,-)$.
2. $\text{Hom}(-,h):\text{Hom}(-,A)\dot{\to }\text{Hom}(-,B)$.

These natural transformations restate the associativity law for composition.

**Remark 39**. The collection of functors from a category **C** to a category **D** and their natural transformations form a *functor category*, denoted **D**^**C**^. Hence, a hom-set from a functor *F* to a functor *G* in **D**^**C**^ is the collection of natural transformation ${\text{Hom}}_{{\text{D}}^{\text{C}}}(F,G)$, also denoted Nat(*F*, *G*).

### Universal constructions

**Definition 40** (Bifunctor). A *bifunctor* is a map *F*: **A** × **B** → **E** such that:

- for each pair of objects (*A*, *B*) in **A** × **B** the maps *F*(*A*, −): **B** → **E** and *F*(−, *B*): **A** → **E** are functors, and
- for each pair of morphisms (*f*: *A* → *C*, *g*: *B* → *D*) in **A** × **B** the following diagram commutes:

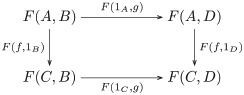
(5)

**Remark 41**. In other words, the functoriality of each argument (first condition) must be jointly compatible (second condition).

**Example 42**. (Bivariate hom-functor). Hom(−, −): **C**^op^ × **C** → **Set**.

**Remark 43**. There is an analogous notion of naturality between bifunctors.

**Definition 44** (Dinatural transformation). Let *F*, *G*: **C**^**op**^ × **C** → **D** be a pair of bifunctors. A *dinatural transformation*, written $\omega :F\ddot{\to }G$, is a family of **D**-morphisms $\left\{\omega_{A}:F(A,A)\to G(A,A)|A\in O(C)\right\}$ such that for each morphism *f*: *A* → *B* in **C** the following hexagon commutes

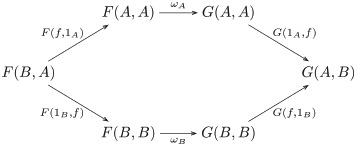
(6)

**Remark 45**. An ordinary natural transformation $\eta :F\dot{\to }G$ is equivalent to a projection of a dinatural transformation: e.g., $\eta \overset{`}{\Pi }:F\circ \overset{`}{\Pi }\ddot{\to }G\circ \overset{`}{\Pi }$, where $\overset{`}{\Pi }$ is a projection functor (see example 29). In this situation, the commutative hexagon for $\eta \overset{`}{\Pi }$ reduces to the commutative square for *η* by effectively ignoring the first variable.

**Definition 46** (Wedge). A *wedge* to a functor *F*: **C**^**op**^ × **C** → **D** is a dinatural transformation $\omega :D\ddot{\to }F$ consisting of a family of **D**-morphisms $\left\{\omega_{A}:D\to F(A,A)|A\in O(C)\right\}$ such that for each morphism *f*: *A* → *B* in **C** the following diagram commutes:

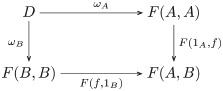
(7)

**Definition 47** (End). The *end* of a functor *F*: **C**^**op**^ × **C** → **D** is a pair (*E*, *ω*) consisting of an object *E* in **D** and a wedge $\omega :E\ddot{\to }F$ such that for every wedge $\beta :Z\ddot{\to }F$ there exists a unique morphism *u*: *Z* → *E* such that *β* = *ω* ∘ *u*, as indicated by the following commutative diagram:

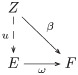
(8)
Object *E* is also denoted *∫*_*A*∈**C**_ *F*(*A*, *A*), or *∫*_**C**_ *F*.

**Remark 48**. An end is a *universal wedge*, i.e. a universal morphism in a category of wedges and wedge morphisms (see definition 49, cf. diagram 8 and diagram 10).

**Definition 49** (Universal morphism). The definition of universal morphism has two forms: (a) primal and (b) dual, which obtains from reversing the directions of arrows in the primal form.

1. *Primal*: Let *F*: **D** → **C** be a functor and *X* an object in **C**. A *universal morphism* from *X* to *F* is a pair (*A*, *ϕ*) consisting of an object *A* in **D** and a morphism *ϕ*: *X* → *F*(*A*) in **C** such that for every object *Y* in **D** and every morphism *f*: *X* → *F*(*Y*) in **C** there exists a unique morphism *u*: *A* → *Y* in **D** such that *f* = *F*(*u*) ∘ *ϕ*, as indicated by commutative diagram

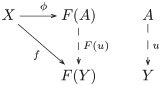
(9)
2. *Dual*: Let *F*: **C** → **D** be a functor and *Y* an object in **D**. A *universal morphism* *from* *F* *to* *Y* is a pair (*B*, *ψ*) consisting of an object *B* in **C** and a morphism *ψ*: *F*(*B*) → *Y* in **D** such that for every object *X* in **C** and every morphism *g*: *F*(*X*) → *Y* in **D** there exists a unique morphism *u*: *X* → *B* in **C** such that *g* = *ψ* ∘ *F*(*u*), as indicated by commutative diagram

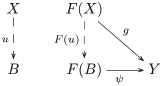
(10)

**Example 50** (Product). A product of *A* and *B* is the universal morphism (*A* × *B*, *π*) from the diagonal functor, Δ, to the pair of objects (*A*, *B*), where $\pi =(\overset{´}{\pi },\overset{`}{\pi })$.

**Proposition 51** (Natural Hom-set). Let *F*, *G*: **C** → **D** be functors. The end of Hom_**D**_(*F*−, *G*−) is the set of natural transformations Nat(*F*, *G*), i.e *∫*_**C**_ Hom_**D**_(*F*−, *G*−) ≅ Nat(*F*, *G*).

*Proof*. The end of this functor restates the naturality condition. Substitution yields the following commutative diagram:

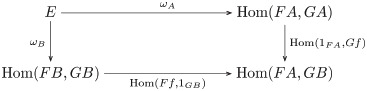
(11)
Commutativity says that for each element *x* ∈ *E* there is a pair of morphisms *ω*_*A*_(*x*) ∈ Hom(*FA*, *GA*) and *ω*_*B*_(*x*) ∈ Hom(*FB*, *GB*), labeled *η*_*A*_ and *η*_*B*_ (respectively), such that *G*(*f*) ∘ *η*_*A*_ = *η*_*B*_ ∘ *F*(*f*) for every morphism *f* in **C**, restating the naturality condition (diagram 1). Thus, *x* identifies with a natural transformation $\eta :F\dot{\to }G$. Universality says that *E* consists of those elements that are necessary and sufficient to identify every natural transformation from *F* to *G*, i.e. *E* ≅ Nat(*F*, *G*).

**Remark 52**. Universal morphisms are *unique up to unique isomorphism*, meaning that for any other universal morphism (*B*′, *ϕ*′) there is one and only one isomorphism, *B* ≅ *B*′, that makes the associated diagram commute. In this regard, universal morphisms are essentially the same, hence referenced as *the* (rather than *a*) universal morphism. The *canonical* universal morphism is the one conventionally given for the situation: e.g., the canoncial product of objects *A* and *B* (example 50) is the universal morphism (*A* × *B*, *π*). However, (*B* × *A*, *π*′), where $\pi^{\prime }=(\overset{`}{\pi },\overset{´}{\pi })$, is also a product of *A* and *B*—there may be more than one isomorphism *A* × *B* ≅ *B* × *A*, but there is only one isomorphism making the associated diagram for products commute: $\overset{`}{\pi }\times \overset{´}{\pi }$.

**Definition 53** (Terminal). In a category **C**, a *terminal object* is an object, denoted 1, such that for every object *Z* in **C** there exists a unique morphism *u*: *Z* → 1.

**Example 54** (Singleton set). The terminal object in **Set** is any singleton set.

**Definition 55** (Initial). In a category **C**, an *initial object* is an object, denoted 0, such that for every object *Z* in **C** there exists a unique morphism *u*: 0 → *Z*.

**Example 56** (Empty set). The initial object in **Set** is the empty set.

### Reconstruction

**Definition 57** (Representable functor). A *representable functor* is a (set-valued) functor *F*: **C** → **Set** that is naturally isomorphic to a hom-functor Hom_**C**_(*A*, −) for an object *A* in **C**, i.e. *α*: Hom(*A*, −) ≅ *F*, as indicated by commutative diagram

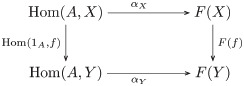
(12)
Functor *F* is said to be *represented* by the pair (*A*, *α*).

**Example 58** (Hom-functors) Hom-functors are representable functors, as they are naturally isomorphic to themselves.

**Proposition 59** (Forgetful functor). *U*: **MSet** → **Set** is a representable functor.

*Proof*. The proof turns on showing that *U* is naturally isomorphic to a hom-functor, specifically Hom_**MSet**_(*M*^*l*^, −). For an M-set representation (*S*, *σ*), we are required to show that Hom_**MSet**_(*M*^*l*^, (*S*, *σ*)) ≅ *S*, i.e., a bijection between the set of equivalent maps, {*f*: *M* → *S*}, and *S*. An equivariant map, *f*, satisfies a commutativity condition (see diagram 4). For the unit, *e* ∈ *M*, commutativity says that *f*(*a*) = *a* ⋅ *f*(*e*). So, for each *a* ∈ *M*, the number of elements *f*(*a*) ∈ *S* is entirely determined by the number of maps *f* ∈ Hom_**MSet**_(*M*^*l*^, (*S*, *σ*)). Hence, Hom_**MSet**_(*M*^*l*^, (*S*, *σ*)) ≅ *S*.

**Remark 60**. Hom_**MSet**_(*M*^*l*^, *M*^*l*^) ≅ *M*. Set (*S*, *σ*) to *M*^*l*^ in proposition 59.

**Proposition 61** (*M*^*l*^). The functor *M*^*l*^ is a representable functor.

*Proof*. The proof follows the reasoning for proposition 59, i.e. we are required to show that *M*^*l*^ is naturally isomorphic to a hom-functor, in this case ${\text{Hom}}_{M^{\text{op}}}(*,-)$. Recall that *M*^*l*^(*) = *M*, so we are required to show that ${\text{Hom}}_{M^{\text{op}}}(*,*)≅M$. In fact, we have ${\text{Hom}}_{M^{\text{op}}}(*,*)=M$ (remark 8).

**Lemma 62** (Yoneda). Let *F*: **C** → **Set** be a set-valued functor, and *A* an object in **C**. The set of natural transformations from the hom-functor Hom_**C**_(*A*, −) to *F* is isomorphic to *F*(*A*), i.e. Nat(Hom_**C**_(*A*, −), *F*) ≅ *F*(*A*).

*Proof*. See [28], theorem 4.2.1.

**Remark 63**. Category **C** is assumed to be *locally small*: all hom-sets in **C** are sets, not proper classes [14].

**Corollary 64** (Embedding). Let *A* and *B* be objects in a (locally small) category **C**. We have Nat(Hom_**C**_(*A*, −), Hom_**C**_(*B*, −)) ≅ Hom_**C**_(*B*, *A*).

*Proof*. Apply Yoneda (lemma 62) with *F* set to Hom_**C**_(*B*, −).

**Theorem 65** (Reconstruction). Let **MSet** be a category of M-set representations for a monoid *M*, and *U*: **MSet** → **Set** the forgetful functor. The end of Hom_**Set**_(*U*−, *U*−) is isomorphic to the monoid *M*, i.e. *∫*_**MSet**_ Hom_**Set**_(*U*−, *U*−) ≅ *M*.

*Proof*. The proof turns on *U* and *M*^*l*^ being representable functors and applications of the Yoneda lemma.

$$\begin{array}{rr} \int_{\text{MSet}}{\text{Hom}}_{\text{Set}}(U-,U-) & \\ ≅ & \left(\text{end}—\text{prop}.\;51\right)\\ \text{Nat}(U,U) & \\ ≅ & \left(\text{representable}—\text{prop}.\;59\right)\\ {\text{Nat}(\text{Hom}}_{\text{MSet}}(M^{l},-),{\text{Hom}}_{\text{MSet}}(M^{l},-)) & \\ ≅ & \left(\text{Yoneda}—\text{cor}.\;64\right)\\ {\text{Hom}}_{\text{MSet}}(M^{l},M^{l}) & \\ = & \left(\text{representable}—\text{prop}.\;61\right)\\ {\text{Nat(Hom}}_{M^{\text{op}}}(*,-),{\text{Hom}}_{M^{\text{op}}}(*,-)) & \\ = & \left(\text{Yoneda}—\text{cor}.\;64\right)\\ {\text{Hom}}_{M^{\text{op}}}(*,*) & \\ = & \left(\text{prop}.\;9\right)\\ M. & \end{array}$$

**Remark 66**. Substituting Hom_**Set**_(*U*−, *U*−) for functor *F* in definition 47 yields commutative diagram

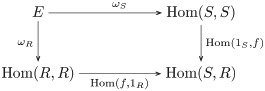
(13)
Commutativity says that for each element *x* ∈ *E* there is a pair of transformations *ω*_*S*_(*x*) ∈ Hom(*S*, *S*) and *ω*_*R*_(*x*) ∈ Hom(*R*, *R*), which we label *σ*_*x*_ and *ρ*_*x*_ (respectively), such that *f* ∘ *σ*_*x*_ = *ρ*_*x*_ ∘ *f*. In other words, *x* picks out a transformation of *S* that corresponds to a transformation of *R*. In this way, *E* corresponds to the acting monoid. (NB. Hom_**MSet**_(*M*^*l*^, *M*^*l*^) ≅ *M* as sets, see remark 60.).

**Remark 67**. The reconstruction theorem for monoids generalizes to a reconstruction theorem for (small) categories, by replacing the monoid *M* with a category **C**. In this situation, the hom-sets of **C** are the actions on sets. The associated representations and equivariant maps form a functor category, **Set**^**C**^, called the permutation representation of **C**, denoted Rep_**Set**_(**C**). The analogous forgetful functor (also called the *fibre functor*) is used to reconstruct the category. Thus, we have the following more general form of the reconstruction theorem for permutation representations of (locally small) categories.

**Theorem 68** (Reconstruction—category). Let Rep_**Set**_(**C**) be the representation permutation category for a (locally small) category **C**, and *F*_*A*_: Rep_**Set**_(**C**) → **Set** the underlying (fibre) functor at object *A* in **C**. We have ${\text{Hom}}_{\text{Se}{\text{t}}^{\text{C}}}(F_{A},F_{B})≅{\text{Hom}}_{\text{C}}(A,B)$.

*Proof* See [13].

**Remark 69**. For comparison, *U* corresponds to the fibre functor evaluated at *, i.e. F_*_, and so we have $\int_{\text{MSet}}{\text{Hom}}_{\text{Set}}(F_{*},F_{*})≅{\text{Hom}}_{M^{\text{op}}}(*,*)=M$.

### Completion

**Definition 70** (Free M-set representation). Let *M* be a monoid with multiplication *μ*, and *S* a set. The *free M-set representation* on *S* is the M-set representation (*M* × *S*, *μ* × 1_*S*_). The action is *μ* × 1_*S*_: *M* × *M* × *S* → *M* × *S*.

**Remark 71**. The free M-set construction is functorial, i.e., we have the functor *F*: **Set** → **MSet**; *S* ↦ (*M* × *S*, *μ* × 1_*S*_), *f* ↦ 1_*M*_ × *f*.

**Definition 72** (Adjunction). An *adjunction* is a pair of functors *F*: **C** → **D** and *G*: **D** → **C** such that for every object *A* in **C** there is a universal morphism from *A* to *G*, i.e. the pair (*F*(*A*), *η*_*A*_), as indicated by the following commutative diagram:

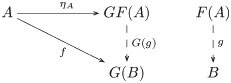
(14)
*F* is called the *left adjoint* to *G*, written *F* ⊣ *G*.

**Remark 73**. Adjoint functors are “pseudo-inverses” but not necessarily actual inverses: the round trip does not return to the original object/morphism, but to an object/morphism that is related to the original by a natural transformation. There are two such natural transformations, $\eta :1_{C}\dot{\to }G\circ F$ and $\epsilon :F\circ G\dot{\to }1_{D}$.

**Example 74** (Free-forgetful). The free M-set functor *F*: **Set** → **MSet** is left adjoint to the forgetful functor *U*: **MSet** → **Set**, i.e. *F* ⊣ *U*, as indicated by the following commutative diagram:

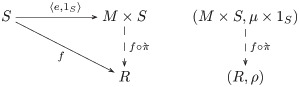
(15)
